# Dolphins and Diabetes: Applying One Health for Breakthrough Discoveries

**DOI:** 10.3389/fendo.2014.00227

**Published:** 2014-12-22

**Authors:** Stephanie Venn-Watson

**Affiliations:** ^1^Translational Medicine and Research Program, National Marine Mammal Foundation, San Diego, CA, USA

**Keywords:** bottlenose dolphin, insulin resistance, marine mammal, metabolic syndrome, one health, type 1 diabetes, type 2 diabetes

The vigilant care of approximately 80 bottlenose dolphins (*Tursiops truncatus*) at the Navy Marine Mammal Program (MMP) has led to this population’s higher annual survival rates, a 10-year longer lifespan, and a higher proportion of dolphins living 30–50 years compared to their wild counterparts (1, 2). The MMP’s unprecedented biobank of tens of thousands of archived clinical samples, collected as part of their care throughout the dolphins’ lives for near a half-century, has been key to improving their health, as well as to discovering the unexpected. During 2007, our clinical research team was reviewing feeding and fasting blood data from thousands of clinical blood samples. Surprisingly, dolphins appeared to readily switch in and out of diabetes-like states. Overnight fasted samples collected in the morning had changes mimicking populations of people with diabetes, including increased glucose, gamma-glutamyl transpeptidase, alkaline phosphatase; and decreased uric acid compared to samples collected from the same dolphins later in the day (3). When dolphins were fed a big meal of fish, they had an insulin-resistant response (sustained postprandial hyperglycemia and hyperinsulinemia) (4). When fed water with 10% dextrose, however, they had an insulin-deficient response (sustained postprandial hyperglycemia with no insulin) (5). Thus, dolphins could switch in and out of both type 2 and type 1 diabetes-like states.

These metabolic switches in dolphins were initially believed to be limited to a natural, non-pathologic state. We now know, however, that dolphins can develop metabolic syndrome, a disease also called prediabetes in humans, which affects one of every three adults in the U.S. (4–10). Interestingly, there is common ground for co-evolved metabolism in dolphins and humans, including shared large brain sizes with high demands for readily available glucose and red blood cells that rapidly transport glucose, a trait once thought to be unique to primates (11, 12). Further, the carnivore hypothesis proposes that insulin resistance in humans evolved to sustain adequate glucose levels when few dietary carbohydrates were available during the Ice Age (13). This evolutionary driver for insulin resistance coincides well with the movement of dolphins from land to sea and their similar dietary shift from a high carbohydrate to high protein diet (14).

This Frontiers in Endocrinology Research Topic, “Marine Mammals as Out-of-the-box Models for Diabetes and Insulin Resistance,” brings together novel science and sage thoughts from multiple disciplines of experts who sought to expand our knowledge of dolphin metabolism, metabolic disease, and their relevance to human health. New science shared within includes identification of case and reference dolphin populations for metabolic syndrome, which open promising doors to discovering risk and protective factors for this disease (5). Dr. Randall Wells et al. review the life history of free-ranging dolphins living in Sarasota Bay, FL, USA to propose potential means to treat and prevent insulin resistance, including diversifying fish species eaten, and grazing or snacking throughout the day and night instead of eating fewer, large meals (15).

Dr. Benjamin Neely et al. dive deeper to assess the potential risk of insulin resistance in dolphins with lower levels of high-weight adiponectin (5, 16). Previous studies in mice and humans have demonstrated the insulin-sensitizing effect of adiponectin and its decreasing beneficial impact in the face of iron overload. Dr. Neeley et al.’s discovery of disrupted associations between adiponectin and glucagon in dolphins with high insulin and iron overload, paired with high sequence similarity of dolphin and human adiponectin, continue to support increasing high-weight adiponectin as a potential therapeutic target for people and other animals with these diseases (16). Dr. Kirsten Eberle et al. use their newly developed, dolphin-specific immunoassays to characterize inflammation present in a 46-year old dolphin with metabolic syndrome and iron overload compared to a dolphin without these diseases (17). Parallels in chronic inflammation among people and dolphins with metabolic syndrome are reported, enhancing the emerging story of chronic inflammation and metabolic perturbations.

Dr. Thomas Schermerhorn describes how the same metabolic response can be normal in carnivores (cats and dolphins) but cause diabetes in humans, providing potential clues as to how insulin resistance may be better controlled in humans (18). A review of northern elephant seal metabolism by Dr. Dorian Houser et al. showcases an extreme version of fasting metabolism in mammals, including their amazing ability to fast for months and sustain a diabetes-like state without developing ketoacidosis (19). Understanding how northern elephant seals can protect themselves against this deadly metabolic state may lead to new approaches in humans with type 1 diabetes. Dr. Sam Ridgway demonstrates dolphins’ lack of an increased respiratory quotient (i.e., oxidative response) while metabolizing glucose, a potential trick that may protect dolphins against the direct, damaging effects of hyperglycemia (20).

This Research Topic also has two articles proposing dolphins, with large volumes of forcefully exhaled breaths from deep lung, as ideal for discovering novel breath-based indicators of metabolism (20, 21). Dolphins have evolved to move large amounts of oxygen from the air into their blood with extreme efficiency using double capillary layers in the lungs. In turn, this trait enables blood-based metabolites to cross over to their breath, providing a gift of clean, compound-rich exhaled breath condensate prime for biomarker discovery. These biomarkers may one day enable non-invasive screening and monitoring of metabolic health in humans and wild cetaceans (22).

The global pandemic of diabetes demands big, game-changing discoveries. Outside traditional scientific laboratories are millions of years of trials that have led to an estimated 8.7 million eukaryotic species on Earth, most of which are in the ocean (23). Today, mechanical engineers are looking to butterflies, bees, and cheetahs for novel ideas to advance machines. Similarly, we can look increasingly to nature for new approaches to cure diseases. By doing so, there comes the benefit of calling upon human health experts to improve animal and environmental health, too. This is the core of the global One Health movement. By taking exceptional care of one together, we can help many. Earth’s 8.7 million species, including the bottlenose dolphin, means 8.7 million chances to make big, positive changes, including better preventing, managing, treating, and curing diabetes. As we move forward, may we actively engage in applying One Health to work together – as physicians, veterinarians, scientists, and biologists – to improve health for all.

## Conflict of Interest Statement

The author declares that the research was conducted in the absence of any commercial or financial relationships that could be construed as a potential conflict of interest.
